# Competition modulates the effects of social comparison on ERP responses during face processing

**DOI:** 10.1093/scan/nsaf005

**Published:** 2025-01-13

**Authors:** Huiyan Lin, Jiafeng Liang

**Affiliations:** Laboratory for Behavioral and Regional Finance, School of National Finance, Guangdong University of Finance, Guangzhou 510521, China; Institute of Applied Psychology, School of Psychology and Entrepreneurship Education, Guangdong University of Finance, Guangzhou 510521, China; School of Education, Guangdong University of Education, Guangzhou 510303, China

**Keywords:** competition, social comparison-related contexts, face perception, P100, N170, LPP

## Abstract

Little is known about the effect of prior social performance feedback on face processing. Our previous study explored how equal and unequal social comparison-related outcomes modulate event-related potential (ERP) responses to subsequently presented faces, where interests between oneself and others were independent (noncompetitive situations). Here, we aimed to extend this investigation by assessing how different unequal social comparison-related outcomes affect face processing under noncompetitive and competitive situations (i.e. a conflict of interest exists between the self and others). To address this issue, 39 participants were exposed to self-related and social comparison-related outcomes, categorized as positive or negative, after performing an attentional task with peers. Rewards and punishments depended on social comparison-related outcomes in the competition condition and on self-related outcomes in the noncompetition condition. ERP results showed that social comparison-related outcomes influenced P100 responses to faces in the self-positive condition. More notably, the effects on N170 responses observed in the noncompetition condition were absent in the competition condition. There was an effect on late positive potential responses only in the competition and self-negative condition. These findings suggest that social comparison-related outcomes influence early face processing irrespective of competition, while competition subsequently disrupts this processing but, later, enhances depending on self-related outcomes.

## Introduction

Faces are crucial in human nonverbal communications, as they can transmit a wide variety of social information about a person. In routine life, faces are often viewed within social contexts, in particular socio-emotional contexts. Accumulated studies have repeatedly investigated whether socio-emotional contexts influence how we interpret and process simultaneously presented faces (e.g. Righart and De Gelder 2006, 2008, Gu et al. 2013, Kato and Takeda 2017, Xu et al. 2017, Lin and Liang 2019). However, the contexts also appear prior to the faces. It is also interesting to understand how prior emotional knowledge influences face processing.

Several event-related potential (ERP) components [i.e. P100, N170, and late positive potentials (LPPs)] have been identified to reflect the contextual effects on face processing. P100, a positive component that starts ∼100 ms after stimulus onset and is distributed over occipital scalp sites, is thought to reflect early perception of a stimulus (e.g. Luck and Hillyard 1994). N170, a face-sensitive component that peaks ∼170 ms after face onset and is distributed over occipito-temporal scalp sites, is thought to be associated with structural encoding of faces (e.g. Eimer and McCarthy 1999, Eimer 2000a, b). In a subsequent time range, the LPP, which is a positive component that starts at ∼500 ms and is largest over parietal scalp sites, has been shown to reflect attentional allocation during emotional evaluations (e.g. Schupp et al. 2000, Leite et al. 2012, Hilgard et al. 2014).

Several studies have utilized these components to explore whether prior socio-emotional stimuli affect neural responses to faces. Specifically, research on the P100 response has shown that this response is more pronounced for target faces following angry expressions compared to neutral ones (Zhang et al. 2018). Additionally, the P100 response is greater for faces that are preceded by negative scenes compared to positive ones (Hietanen and Astikainen 2013). These findings suggest that early face perception is heightened by preceding negative contexts.

Subsequently, the N170 response was found to be stronger to faces preceded by angry vocal expressions compared to those preceded by neutral expressions (Lin and Liang 2023). However, two other studies reported smaller N170 responses to target faces following contextually negative facial expressions than to those following contextually neutral expressions (Furl et al. 2007, Lin et al. 2015b). Additionally, Diéguez-Risco et al. (2015) found larger N170 responses to positive and negative faces that were preceded by emotionally incongruent compared to congruent sentences, while the effect was reversed when relevant faces were preceded by social scenes (Hietanen and Astikainen 2013) and expectancy cues (Lin et al. 2015a). These findings indicate that prior positive and negative contexts may influence the structural encoding of a face. However, the direction of the effects appears to depend on experimental design and stimulus categories.

Regarding the LPP, studies have shown that the response is larger to positive and negative faces preceded by emotionally incongruent cues (Lin et al. 2015a, 2016, Liang et al. 2024), sentences (Diéguez-Risco et al. 2015), and scenes (Hietanen and Astikainen 2013) compared to congruent ones. This suggests that high arousing and salient contexts enlarge LPP responses. However, Lin et al. (2017) demonstrated that high arousing contextual facial expressions (e.g. surprised expressions) reduced LPP responses to subsequent target faces. These findings might suggest that emotional arousal influences face evaluations, depending on experimental design.

Moreover, several studies have demonstrated that prior negative and/or high arousing performance feedback regarding an individual influences ERP responses to that individual’s face. For example, Matyjek et al. (2021) reported that preceding negative behaviours associated with an individual led to larger P100 responses to their face. Additionally, it has reported that the N170 response is greater to faces whose identities have previously received negative evaluations compared to neutral ones (Krasowski et al. 2021, Schindler et al. 2021). These studies also revealed increased LPP responses to faces linked with negative evaluations, particularly when the task required recalling evaluative information. Additionally, Breton et al. (2014) reported increased LPP responses to faces whose identities had received high social ranks compared to medium and low ones, as well as low ranks compared to medium ones.

Recently, our study (Lin and Liang 2024a) explored the effect of preceding performance feedback by investigating the effects of social comparison-related outcomes on ERP responses during face perception. Social comparison, which refers to comparing one’s own attributes (e.g. abilities and performance outcomes) with those of others (Festinger 1954), alters emotional value of the outcomes of one’s performance depending on the outcomes of others (Collins 1996, Wheeler et al. 1997, Mussweiler et al. 2004, Zell et al. 2020, Lin and Liang 2021c, Lin et al. 2023). For instance, an outcome superior to others (i.e. positive social comparison-related outcomes) typically triggers positive emotions, such as happiness (e.g. Suls et al. 2002) and pride (e.g. Smith 2000). Conversely, an inferior outcome (i.e. negative social comparison-related outcomes) often leads to negative emotions, including envy (e.g. van de Ven and Zeelenberg 2020, Lin and Liang 2021a, b), threat (e.g. Mendes et al. 2001), and shame (e.g. Smith 2000).

In our previous study (Lin and Liang 2024a), participants were told that they and a peer each engaged in a monetary game. Participants were presented with both their own and the peer’s positive or negative outcomes (i.e. monetary gain or loss). Following this, participants were shown the face of the peer with whom they had just been paired. The findings revealed that peer’s positive outcomes, compared to negative outcomes, led to more negative N170 responses to the peer’s face when participants encountered negative outcomes themselves. This suggests that negative social comparison-related outcomes affect the structural encoding of faces. Additionally, the LPP response was generally weaker to peers’ faces when the peers had received positive outcomes compared to negative outcomes. This implies that social outcomes, rather than social comparison-related outcomes, influence face evaluations.

Note that our previous study (Lin and Liang 2024a) primarily investigated the effects between unequal (e.g. self-negative and other positive) and equal social comparison-related outcomes (e.g. self-negative and other negative). However, it remains unknown whether face processing is modulated by different types of unequal social comparison-related outcomes (e.g. positive and negative ones). Given that both prior positive and negative contexts have been shown to influence the abovementioned ERP responses to faces, those responses might also reflect the difference between positive and negative social comparison-related outcomes.

More importantly, in our previous study (Lin and Liang 2024a), rewards and punishments were based on participant’s performance and an individual’s interests were independent of those of others (i.e. noncompetitive circumstances). In social circumstances, however, rewards and punishments are often linked with performances involving social comparison, for instance, individuals are rewarded when their performance surpasses that of others and punished when their performance falls short (competitive circumstances; e.g. Garcia et al. 2013, Tor and Garcia 2023). It remains unclear whether the effects of social comparison-related outcomes on face perception differ between competitive and noncompetitive circumstances. Nevertheless, competition is believed to enhance the processing of social comparison-related outcomes (Garcia et al. 2013, Lin and Liang 2024b). The heightened processing might apply for faces, leading to altered effects of social comparison-related outcomes on ERP responses to faces. Overall, exploring effects of positive and negative social comparison-related outcomes depending on competition could enhance our understanding of how social performance feedback influences face processing in broader social situations.

Accordingly, the present study aimed to investigate whether positive and negative social comparison-related outcomes influence ERP responses during the processing of subsequently presented faces relying on competition. To address this issue, participants were presented with either positive or negative self-related outcomes and social comparison-related outcomes after completing an attentional task with several peers. In the noncompetition condition, rewards and punishments were based on self-related outcomes, while in competition condition, they were determined by social comparison-related outcomes. Subsequently, participants were shown a facial photo that was said to have been taken from their peers.

Based on our previous study (Lin and Liang 2024a), we predicted that social comparison-related outcomes would influence N170 responses but not LPP responses to faces in noncompetitive circumstances. Furthermore, in noncompetitive situations, social comparison-related outcomes are irrelevant to an individual’s interest. This irrelevance might diminish concerns regarding social comparison-related outcomes (Tor and Garcia 2023), particularly those that cannot bring about social rewards (e.g. nonpositive outcomes), resulting in reduced N170 responses. In contrast, competition could enhance the processing of social comparison-related outcomes overall (Garcia et al. 2013, Lin and Liang 2024b), potentially diminishing the differential processing between distinctive social comparison-related outcomes. Therefore, our first hypothesis posited that positive compared to negative social comparison-related outcomes would evoke larger N170 responses to subsequently presented faces in the noncompetition condition, which would be absent in the competition condition.

In contrast to the N170 time range, individuals within the LPP time range exhibit greater flexibility in adjusting their attention. This allows them to potentially enhance their focus on the most arousing stimuli. In the competition condition, the most arousing outcome was the self-negative and social-positive one, which indicated that participants were rewarded despite not performing well. Accordingly, our second hypothesis posits that positive social comparison-related outcomes would enlarge LPP responses specifically in the competition and self-negative condition. However, due to the limited literature on this topic, we did not have a clear hypothesis regarding the P100 effects of social comparison-related outcomes in relation to competition.

## Method

### Participants

Forty-one undergraduate students were recruited as participants. Two participants were excluded due to excessive electroencephalogram (EEG) artefacts. This led to a final sample of 39 participants (24 women; 18–22 years, *M* = 19.35, SD = 0.78). Our previous study revealed a small to medium effect size for the interaction between competition and social comparison-related outcomes, as well as for the interaction among competition, social comparison-related and self-related outcomes (Lin and Liang 2024b). We therefore considered whether the current sample size could also achieve a similar effect size. To assess this, a sensitivity power analysis was conducted using G*power 3.1.7 (Faul et al. 2007). The results revealed that the present sample size had a power of 80% to detect a small to medium effect size (*f* = 0.15–0.19) for the abovementioned 2-/3-way interactions. All participants were right-handed, had normal or corrected-to-normal vision, and did not report neurological illness or a history. All participants provided written informed consent, which was in accordance with standard ethical guidelines from the Declaration of Helsinki. The study was approved by the Academic Committee of School of National Finance, Guangdong University of Finance.

### Stimuli

The stimuli included 42 neutral faces (21 women and 21 men). Of these, forty faces (20 women and 20 men), which were used in the test phase, were obtained from the Chinese Academy of Sciences’ Pose, Expression, Accessory and Lighting Large-Scale Chinese Face Database (Gao et al. 2007). The remaining two faces, which served as practice stimuli, were taken from the experimenters. Facial pictures were cropped similarly around the face outline and centred, so that eyes, noses, and mouths were located at similar positions. For all pictures, external features (e.g. neck, shoulder, distant hair, and jewellery) were removed and the size was adjusted into 6.75 cm × 9 cm (horizontal × vertical). The background colour was set to black. Each facial stimulus (excluding the stimuli presented in the practice) was presented once in each experimental condition.

### Procedure

Prior to the experiment, participants were informed that they would perform an attentional task and obtain or lose 5 RMB according to self-related or social comparison-related outcomes (see the following paragraphs for details). Participants were informed that the overall compensation would be based on the general gain or loss for all trials, entailing the addition or subtraction of the basic compensation of 30 RMB, respectively [e.g. if participants generally gain 10 RMB for all trials in the attentional task, they would receive an overall compensation of 40 (i.e. 30 + 10) RMB; otherwise, if they generally lose 10 RMB, they would receive an overall compensation of 20 (i.e. 30–10) RMB]. In fact, the general gain or loss was randomized by the computers, ranging from −10 to 10 RMB. Nevertheless, to ensure the principle of voluntary participation, participants were also told that the compensation provided to them were noncoercive.

**Figure 1.**
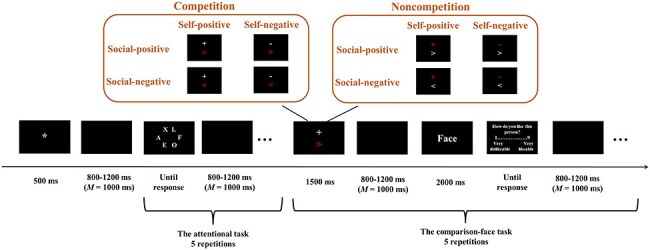
Schematic illustration of the experimental procedure.

As illustrated in Fig. 1, a white fixation point (“*”) was firstly displayed for 500 ms. After showing a blank screen between 800 and 1200 ms (*M* = 1000 ms), there was an attentional task that was repeated 5 times. For each trial of this task, participants had to indicate whether the target letter “F” or “D” appeared among six letters [more details could be found in our previous study (Lin and Liang 2024b)]. A blank screen was presented for a random duration between 800 and 1200 ms (*M* = 1000 ms) after every two trials of the attentional task.

Subsequently, a comparison face task was presented 5 times. In this task, two symbols were displayed on the upper and lower sides of the screen. For half of the participants, the “+” or “−” symbol appeared on the upper side, while the “>” or “<” symbol was shown on the lower side. The positions of the symbols were reversed for the other half of the participants.

The “+” and “−” symbols represented self-related positive and negative outcomes, respectively. Participants were informed that the valence of the outcome depended on the difference between their averaged performances in the current five trials and those in several preceding trials. It was emphasized to participants that the preceding trials were selected randomly by a computer, which meant that self-related outcomes could sometimes be surprising, e.g. outcomes might be positive even if a participant made an incorrect and slow response or negative if the response was correct and fast. This emphasis was intended to enhance participants’ belief in the authenticity of the outcome. In reality, however, the computer did not compare participants’ performances in the current trials with those from preceding trials and all performance outcomes were presented in a randomized order.

The “>” and “<” symbols indicated positive and negative social comparison-related outcomes, respectively. Participants were informed that the experimenters had previously recruited a large number of undergraduate and postgraduate students from other universities or colleges to perform the same attention task. Participants were told that the valence of social comparison-related outcomes was based on outcome differences between the participants themselves and another student. Participants were told that in each trial, their averaged performance over the current five trials of the attentional task would be compared with the averaged performance of another student who had also completed the same five trials. In reality, however, there were no students, and the social comparison-related outcomes were presented in a randomized order.

To understand the influence of competition, participants in the competition condition were told that the “>” and “<” symbols were depicted in red and rewards and punishments depended on social comparison-related outcomes (i.e. the attainment of positive social comparison-related outcomes would result in a gain of 5 RMB, while negative outcomes would lead to a loss of 5 RMB). In the noncompetition condition, the participants were informed that the “+” and “−” symbols were depicted in red and rewards and punishments were based on their own performance outcomes. The sequence of the competition and noncompetition conditions was randomized across participants.

Following another blank screen between 800 and 1200 ms (*M* = 1000 ms), a face was presented at the centre of the screen for 2000 ms. Participants were told that the person was someone they had just played with. After viewing the face, participants were asked to rate to which they liked the person on a 9-point scale (1 = very dislikeable, 9 = very likeable) by pressing the corresponding number on the number keyboard using their right hand. There was no time limit for the ratings. There was another blank screen presented for 800–1200 ms (*M* = 1000 ms).

There were 40 trials for each experimental condition, resulting in a total of 320 trials [40 trials × 2 competition conditions (competition vs. noncompetition) × 2 self-related outcomes (positive vs. negative) × 2 social comparison-related outcomes (positive vs. negative)]. Prior to the experiment, there were eight practice trials to familiarize with the experimental procedure. During the practice session, participants were told that their task performance would be recorded and used in the actual task. However, the self-related and social comparison-related outcomes were presented in a randomized order, and the facial pictures were taken from the experimenters. The experiment (including practice and preparations) lasted ∼1.5 h.

### Behavioural recordings and preprocessing

Participants’ ratings of the extent to which they liked the person they played with (i.e. liking ratings) were recorded for each trial. The ratings were then averaged separately for each experimental condition.

### EEG recording and preprocessing

Continuous EEGs were recorded using two 32-channel BrainAmp amplifiers (Brain Products GmbH, Munich, Germany). Ag/AgCl electrodes were placed on the scalp by using an EasyCap electrode system (EASYCAP GmbH, Herrsching-Breitbrunn, Germany) based on the 10-20 system. The AFz electrode was used as the ground electrode. All other electrodes were referenced to the FCz electrode during data collection. The horizontal electrooculogram (EOG) was recorded from two electrodes at the outer canthi of both eyes, and the vertical EOG was recorded in a bipolar fashion from two electrodes that were on the upper and lower sides of the right eye to monitor eye blinks and movements. The sample rate was 1000 Hz, and band-pass filtering (0.05–100 Hz) was applied with a 50-Hz notch filter online. Impedances were below 10 kΩ for all recordings.

Offline, EEG data were processed using BrainVision Analyzer 2.0 software (Brain Products GmbH, Munich, Germany). Ocular movements were inspected and removed from the EEG data using the algorithm by Gratton et al. (1983). The EEG data were then segmented into 2200 ms epochs from −200 to 2000 ms relative to the onset of facial stimuli, with the first 200 ms epoch used for baseline correction. Artefact rejection was performed with an amplitude threshold of 100 μV, a gradient criterion of 50 μV/ms, and lowest activity criterion of 0.5 μV per 100 ms. Artefact-free trials were then averaged from each channel and experimental condition. Averaged ERPs were low-passed filtered at 30 Hz (24 db/oct, zero phase shift). Finally, ERPs were recomputed to an average reference excluding vertical and horizontal EOG channels. In general, 88.85% of trials were retained.

ERPs were quantified by using the peak amplitudes of the P100 (115–155 ms) and N170 (155–195 ms), and the mean amplitude of the LPP (500–1000 ms). The P100 was measured at the occipital electrodes (i.e. O1, Oz, and O2; PO7 and PO8), the N170 was measured at the parietal-occipital electrodes (i.e. P7, P8, PO7, and PO8), and the LPP was measured at the central-parietal electrodes (i.e. CP3, CPz, and CP4). The time windows for the P100 and N170 were selected based on the peak latency identified in the grand waveforms across all experimental conditions (137 and 175 ms, respectively). The time window for the LPP and the electrodes of interest for all components were selected based on visual inspection of grand waveforms and topography maps and previous studies (e.g. Breton et al. 2014, Krasowski et al. 2021, Matyjek et al. 2021, Schindler et al. 2021, Lin and Liang 2024a).

### Data analysis

Averaged liking ratings were performed using a 2 × 2 × 2 repeated measures analyses of variance (ANOVA) with competition (competition vs. noncompetition), self-related outcomes (positive vs. negative), and social comparison-related outcomes (positive vs. negative) as within-subject factors. In terms of ERP data, the amplitudes of each component were averaged for all electrodes of interest. The amplitudes of these components were assessed by using the abovementioned 2 × 2 × 2 ANOVA.

## Results

### Liking ratings

ANOVA revealed main effects of self-related outcomes [*F*(1, 38) = 10.95, *P* = .002, .224] and social comparison-related outcomes [*F*(1, 38) = 19.34, *P* < .001, $\eta _{\mathrm{p}}^2$ = 0.337]. Liking ratings were generally higher for faces in the self-positive condition than for the faces in the self-negative condition, as well as for faces in the social-positive condition than for the faces in the social-negative condition.

The 2-way interaction between competition and self-related outcomes was significant [*F*(1, 38) = 13.57, *P* = .001, $\eta _{\mathrm{p}}^2$ = 0.263]. Further analysis showed that the effect of self-related outcomes was significant only under the noncompetition condition [*F*(1, 38) = 13.27, *P* = .001, ${{\eta }}_{\mathrm{p}}^2$ = 0.259], but not in the competition condition (*P* = .458).

**Figure 2.**
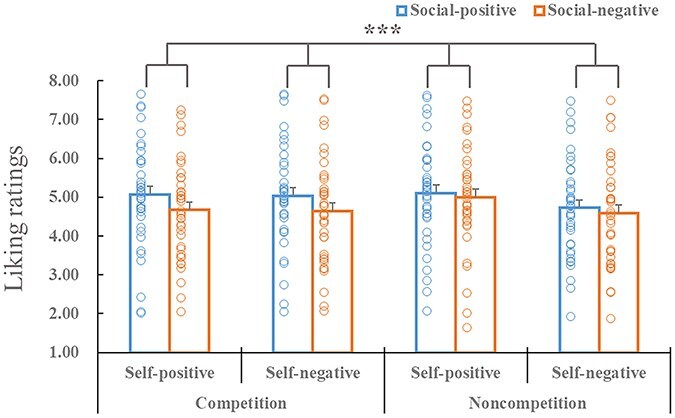
Mean liking ratings and their SEs (vertical lines) for all the experimental conditions. The dots indicate liking ratings for each participant in each condition. The “***” symbol indicates *P *< .001.

There was also a 2-way interaction between competition and social comparison-related outcomes [*F*(1, 38) = 7.52, *P* = .009, ${{\eta }}_{\mathrm{p}}^2$ = 0.165; Fig. 2]. Further analysis revealed that the effect of social comparison-related outcomes was present in both the competition and noncompetition conditions, whereas this effect was larger in the competition condition [*F*(1, 38) = 14.51, *P* < .001, ${{\eta }}_{\mathrm{p}}^2$ = 0.276] than in the noncompetition condition [*F*(1, 38) = 13.63, *P* = .001, ${{\eta }}_{\mathrm{p}}^2$ = 0.264]. Other main effects or interactions were not significant (*P* ≥ .637).

### ERP data

#### P100 responses

**Figure 3.**
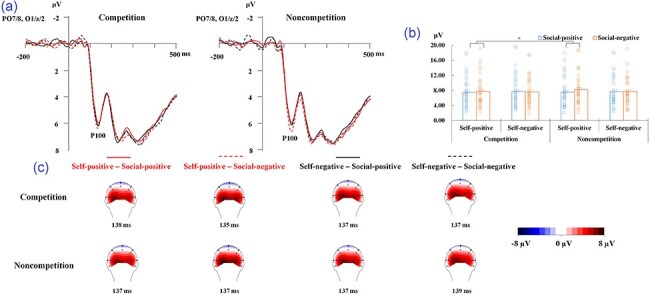
(a) ERP waveforms at electrodes of interest (O1, Oz, O2, PO7, and PO8 were pooled) for all the experimental conditions. (b) Peak amplitude (μV) and SEs and scatterplots of the P100 for all the experimental conditions. The “*” symbol indicates *P* < .05. (c) Topography maps show the peak amplitudes (μV) of the P100 component for all the experimental conditions.

The analysis only revealed an interaction between self-related outcomes and social comparison-related outcomes [*F*(1, 38) = 4.11, *P* = .0497, ${{\eta }}_{\mathrm{p}}^2$ = 0.098; Fig. 3]. Further analysis showed that the P100 response was stronger to faces in the social-negative condition compared to those in the social-positive condition under the self-positive condition [*F*(1, 38) = 5.36, *P* = .026, ${{\eta }}_{\mathrm{p}}^2$ = 0.124], whereas the effect of social comparison-related outcomes was not significant under the self-negative condition (*P* = .955). No any other main effects or interactions were significant (*P* ≥ .117).

#### N170 responses

**Figure 4.**
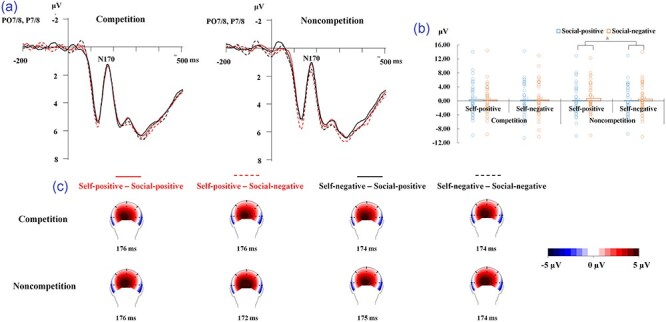
(a) Grand averaged ERP waveforms by pooling the P7, PO7, P8, and PO8 electrodes for all the experimental conditions. (b) Bar charts for means and SEs and scatterplots of the N170 peak amplitudes in different experimental conditions. The “*” symbol indicates *P* < .05. (c) Topography maps based on the peak amplitudes (μV) of the N170 component.

The analysis revealed a main effect of social comparison-related outcomes [*F*(1, 38) = 6.13, *P* = .018, ${{\eta }}_{\mathrm{p}}^2$ = 0.139], which was further quantified by a 2-way interaction between competition and social comparison-related outcomes [*F*(1, 38) = 8.28, *P* = .007, ${{\eta }}_{\mathrm{p}}^2$ = 0.179; Fig. 4]. In line with Hypothesis 1, further analysis revealed a more negative response to faces in the social-positive condition compared to those in the social-negative condition under the noncompetition condition [*F*(1, 38) = 11.56, *P* = .002, .233], but the effect of social comparison-related outcomes was not significant in the competition condition (*P* = .918). There were not any other significant main effects or interactions (*P* ≥ .475).

#### LPP responses

**Figure 5.**
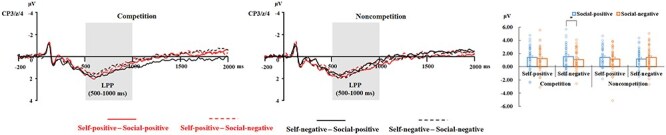
The left and middle panels: ERP waveforms for each experimental condition at electrodes of interest (CP3, CPz, and CP4 were pooled). Shaded areas represent the time windows for the LPP component (500–1000 ms). The right panel: Mean amplitudes (μV) on the LPP and SEs and scatterplots of the LPP amplitudes in each condition. The “*” symbol indicates *P* < .05.

**Figure 6.**
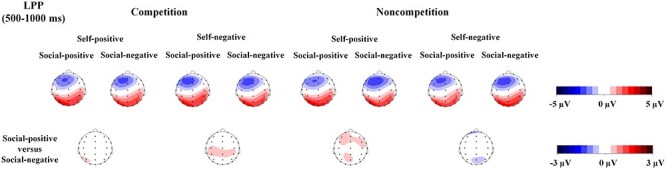
Topography maps based on the mean amplitudes (μV) of the LPP component (500–1000 ms) for all the experimental conditions and for the differences between the social-positive and social-negative conditions for each level of combinations of competition and self-related outcomes.

ANOVA only revealed a 3-way interaction among competition, self-related outcomes, and social comparison-related outcomes [*F*(1, 38) = 4.36, *P* = .044, ${{\eta }}_{\mathrm{p}}^2$ = 0.103; Figs 5 and 6]. Separate analyses conducted for each level of competition did not reveal any interaction between self-related outcomes and social comparison-related outcomes, in both the noncompetition (*P* ≥ .117) and competition conditions (*P* = .237). In the competition condition, the lack of a significant interaction can be attributed to the larger LPP response observed for faces in the social-positive condition compared to those in the social-negative condition [*F*(1, 38) = 6.56, *P* = .015, ${{\eta }}_{\mathrm{p}}^2$ = 0.147]. This finding does not fully align with Hypothesis 2.

To further validate Hypothesis 2, which focuses on the effects of social comparison-related outcomes specifically in the self-negative condition, we employed an alternative approach to address the 3-way interaction. This involves analysing the 2-way interaction between competition and social comparison-related outcomes at each level of self-related outcomes. The findings revealed a significant interaction only in the self-negative condition [*F*(1, 38) = 7.94, *P* = .008, ${{\eta }}_{\mathrm{p}}^2$ = 0.173], but not in the self-positive condition (*P* = .755). Consistent with Hypothesis 2, further analysis of the interaction in the self-negative condition revealed more positive responses to faces associated with social-positive outcomes compared to those linked with social-negative outcomes under the competition condition [*F*(1, 38) = 7.11, *P* = .011, ${{\eta }}_{\mathrm{p}}^2$ = 0.158], whereas the effect of social comparison-related outcomes was not significant in the noncompetition condition (*P* = .199). Other main effects or 2-way interactions were not significant (*P* ≥ .090).

As is shown in Fig. 6, it seemed that there was an effect of social comparison-related outcomes in the noncompetition and self-positive condition over frontal and frontocentral scalp sites. Therefore, we tried to average the amplitudes separately for frontal (F3, Fz, and F4) and frontocentral electrodes (FC3, FCz, and FC4) and performed the same ANOVA separately on the frontal and frontocentral amplitudes of LPP. However, the analysis did not reveal any interactions between social comparison-related outcomes and other factors (all *P* values > .240).

## Discussion

The present study investigated whether competition influenced the effect of social comparison-related context on ERP responses during face perception. Results showed that social-negative compared to social-positive outcomes elicited a larger P100 response to faces in the self-positive condition, irrespective of competition. More notably, social-positive compared to social-negative outcomes resulted in a more negative N170 response to subsequently presented faces under the noncompetition condition, an effect that was not observed under the competition condition. Additionally, faces preceded by social-positive outcomes elicited a larger LPP response compared to those preceded by social-negative outcomes, specifically in the competition and self-negative condition. The findings might suggest that prior positive and negative social comparison-related outcomes have differential influences on face processing across various time ranges, depending on self-related outcomes and, importantly, the presence of competition.

The P100 is thought to reflect early stimulus perception (e.g. Luck and Hillyard 1994). Research indicates that prior exposure of negative stimuli, e.g. faces and scenes (Hietanen and Astikainen 2013, Zhang et al. 2018), as well as negative social knowledge (Matyjek et al. 2021), enlarges the P100 response to faces. This suggests that negative social contexts exert a more significant influence on early face perception. Accordingly, the current findings suggest that negative social comparison-related outcomes may strengthen early face perception more substantially than positive outcomes, particularly in the self-positive condition.

It has been suggested that negative social comparison-related outcomes elicit negative emotions, such as envy (e.g. van de Ven and Zeelenberg 2020), threat (e.g. Mendes et al. 2001), and shame (e.g. Smith 2000). Envy and threat prompt individuals heighten their vigilance to those they perceive as superior (e.g. Koster et al. 2004, Hill et al. 2011). In contrast, feelings of shame lead individuals to avoid engaging with superior persons (Schmader and Lickel 2006). In the present study, participants who obtained negative social comparison-related outcomes might have generally felt envy and threat. However, those in the self-negative condition might have felt more shame than those in the self-positive condition. This increased sense of shame could reduce attention to the faces of superior individuals, leading to a diminished impact of social comparison-related outcomes in the self-negative condition.

In later time ranges, the N170 is associated with early configural and structural perception of a face (e.g. Eimer and McCarthy 1999, Eimer 2000a, b) and the LPP with emotional evaluations (e.g. Schupp et al. 2000, Leite et al. 2012, Hilgard et al. 2014). The current findings might suggest that competition weakens distinct structural encoding of faces associated with positive and negative social comparison-related outcomes, while it strengthens differential evaluations of relevant faces in the self-negative condition.

As mentioned earlier, the N170 and LPP reflect not only negative but also positive contextual stimuli. Accordingly, for the current study, not only negative but also positive social comparison-related outcomes elicited N170 and LPP responses. It has been suggested that individuals who obtain positive social comparison-related outcomes express pride to inferior others (e.g. Smith 2000). This expression might increase attention to others’ faces, even overpassing attention in the social-negative condition, and thus enhance structural encoding and emotional evaluation of relevant faces.

Nevertheless, increased responses to faces associated with positive social comparison-related outcomes, compared to those linked with negative outcomes, were observed only in the noncompetition condition during the N170 time range, but in the competition and self-negative condition in the LPP time range. In the competition condition, social comparison-related outcomes were particularly relevant to self-interest, which might strengthen attention to social comparison-related outcomes (Garcia et al. 2013, Lin and Liang 2024b). The N170 reflects automatic attention to faces (Cauquil et al. 2000), and during this stage, brains might fail to adjust attention flexibly. In this case, enhanced attention could occur regardless of outcome valence, leading to a reduction in the effect of social comparison-related outcomes on face perception.

In contrast, the LPP is associated with top-down attentional modulation (Hietanen et al. 2014). During this time range, individuals might initially direct their attention toward high arousing faces by integrating various contextual factors, including competition and self-related and social comparison-related outcomes. Among all outcomes in the competition condition, the most arousing outcome was the self-negative and social-positive outcome, where participants did not perform well but could still receive a reward. This outcome might have heightened attention to faces, thereby amplifying the effect of social comparison-related outcomes in the competition and self-negative condition.

Moreover, the current findings revealed that the effects of social comparison-related outcomes on face processing were modulated only by self-related outcomes in P100 time ranges, competition in N170 time ranges, and both competition and self-related outcomes in LPP time ranges. The findings suggest that during face processing, brains integrate social comparison-related outcomes firstly with self-related outcomes, followed by competition, and finally with both competition and self-related outcomes. In the present study, participants were instructed to make a social comparison with facial identities. This association between faces and social comparison-related outcomes might lead to the prioritization of processing these outcomes during face processing. Although self-related outcomes and competition environments are not directly related to faces, they are thought to modulate evaluations of social comparison-related outcomes (e.g. Lin et al. 2023, Lin and Liang 2024b), potentially influencing face processing. Nevertheless, it is possible that brains cannot initially allocate attentional resources to process both face-irrelevant contexts in early time ranges but can do so in later time ranges (Lin and Liang 2024b). This could result in the effect of social comparison-related outcomes being modulated by either of the face-irrelevant contexts in early time ranges but by both contexts at later ones. Additionally, the earlier modulation of self-related outcomes compared to competition might be because self-related outcomes are crucial for evaluating the performance of facial identities, thus becoming associated with relevant faces.

The current findings align with our previous study (Lin and Liang 2024a), which found no effect of social comparison-related outcomes on LPP responses to faces in a noncompetition setting. Different from the current findings, however, our previous study revealed that negative social comparison-related outcomes amplified N170 responses to the faces of others. This investigation focused on the distinction between negative and equal social comparison-related outcomes, perceived as negative and neutral, respectively. Negative social feedback compared to neutral ones has been linked to heightened N170 responses to subsequent faces (Krasowski et al. 2021, Schindler et al. 2021). However, our present study investigated the contrast between unequal positive and negative outcomes. The extent of the N170 response difference between these two outcomes is dependent on the specific experimental design.

Additionally, behavioural findings revealed that faces in the social-positive condition were rated as more likeable than those in the social-negative condition. This effect of social comparison-related outcomes was more pronounced in the competition condition compared to the noncompetition condition. Theories and empirical studies on evaluative conditioning have suggested that neutral stimuli (e.g. faces) can acquire emotional meanings even though they are merely paired with relevant emotional stimuli (Hofmann et al. 2010, Hütter 2022, Moran et al. 2023). Moreover, competition is known to intensify the processing of outcomes related to social comparisons, including emotional evaluations (Lin and Liang 2024b). Such strengths might also extend to face evaluations, resulting in more significant effects of social comparison-related outcomes on liking ratings of faces.

Overall, several studies have suggested that social feedback influences face processing (Breton et al. 2014, Krasowski et al. 2021, Matyjek et al. 2021, Schindler et al. 2021). Our previous study (Lin and Liang 2024a) highlighted that unequal negative social comparison-related outcomes, compared to equal ones, affected early face perception in noncompetition circumstances. The current study offered new insights, revealing that both unequal positive and negative social comparison-related outcomes affected face processing in both early and late time ranges. Crucially, the present findings also provided new evidence that the effects of social comparison-related outcomes are firstly modulated by self-related outcomes, then by competition, and ultimately by a combination of both factors. These findings might further illustrate that both positive and negative social feedback can influence face processing, contingent on face-irrelevant social (e.g. competition) and nonsocial (e.g. self-related outcomes) factors.

Additionally, the current findings have implications for person-to-person interactions in real life. The current study suggested that comparisons with others can alter perceptions and evaluations during subsequent presentations of other’s faces based on competitive circumstances. Such alternations may persist over time, potentially influencing future social interactions and attitudes toward relevant individuals (Breton et al. 2014, Krasowski et al. 2021, Matyjek et al. 2021, Schindler et al. 2021). Moreover, social feedback associated with specific faces can generalize to other faces perceived as similar (Lin et al. 2024). Therefore, the influence of social comparison-related outcomes observed in the present study might also extend to other faces that resemble those of the comparators.

Finally, we would like to mention some of the limitations of the current study and make suggestions for upcoming studies. Previous studies have demonstrated that social comparison-related outcomes influence face memory (Sugimoto et al. 2021, Lin and Liang 2024a). However, it remains unclear whether these effects are modulated by competitive circumstances. Future studies should further explore this issue. Additionally, social comparison can also occur in cooperation situations, such as when rewards are contingent upon both individual and group performance (Valt et al. 2020). Future studies could investigate whether social comparison influences face processing in these cooperation contexts.

## Conclusion

The current study revealed that the P100 response was larger to faces preceded by negative social comparison-related outcomes compared to positive ones when one received a positive outcome. More importantly, the larger N170 responses to faces preceded by positive social comparison-related outcomes compared to negative ones, observed in the noncompetition condition, was absent in the competition condition. Additionally, the LPP response was larger for faces following positive social comparison-related outcomes compared to negative outcomes, only when one received a negative outcome in the competition condition. Taken together, these findings imply that social comparison-related contexts influence face perceptions in very early time ranges, irrespective of competition. Competition appears to weaken the contextual effects in subsequent time ranges and later strengthen them depending on self-related outcomes.

## Data Availability

The data underlying this article will be shared on reasonable request to the corresponding author.

## References

[R1] Breton A, Jerbi K, Henaff MA et al.. Face the hierarchy: ERP and oscillatory brain responses in social rank processing. *PLoS One* 2014;9:e91451. doi: 10.1371/journal.pone.0091451 PMC3951356 24622288

[R2] Cauquil AS, Edmonds GE, Taylor MJ. Is the face-sensitive N170 the only ERP not affected by selective attention?. *Neuroreport* 2000;11:2167–71. doi: 10.1097/00001756-200007140-00021 10923664

[R3] Collins RL . For better or worse: the impact of upward social comparison on self-evaluations. *Psychol Bull* 1996;119:51–69. doi: 10.1037/0033-2909.119.1.51

[R4] Diéguez-Risco T, Aguado L, Albert J et al.. Judging emotional congruency: explicit attention to situational context modulates processing of facial expressions of emotion. *Biol Psychol* 2015;112:27–38. doi: 10.1016/j.biopsycho.2015.09.012 26450006

[R5] Eimer M . Effects of face inversion on the structural encoding and recognition of faces: evidence from event-related brain potentials. *Cognit Brain Res* 2000a;10:145–58. doi: 10.1016/S0926-6410(00)00038-0 10978702

[R6] Eimer M . The face-specific N170 component reflects late stages in the structural encoding of faces. *Neuroreport* 2000b;11:2319–24. doi: 10.1097/00001756-200007140-00050 10923693

[R7] Eimer M, McCarthy RA. Prosopagnosia and structural encoding of faces: evidence from event-related potentials. *Neuroreport* 1999;10:255–59. doi: 10.1097/00001756-199902050-00010 10203318

[R8] Faul F, Erdfelder E, Lang AG et al.. G* Power 3: a flexible statistical power analysis program for the social, behavioral, and biomedical sciences. *Behav Res Methods* 2007;39:175–91. doi: 10.3758/BF03193146 17695343

[R9] Festinger L . A theory of social comparison processes. *Human Relations* 1954;7:117–40. doi: 10.1177/001872675400700202

[R10] Furl N, Van Rijsbergen NJ, Treves A et al.. Experience-dependent coding of facial expression in superior temporal sulcus. *Proc Natl Acad Sci USA* 2007;104:13485–89. doi: 10.1073/pnas.0702548104 17684100 PMC1948923

[R11] Gao W, Cao B, Shan S et al.. The CAS-PEAL large-scale Chinese face database and baseline evaluations. *IEEE Trans Syst Man Cyber-Part A: Syst Humans* 2007;38:149–61.

[R12] Garcia SM, Tor A, Schiff TM. The psychology of competition: a social comparison perspective. *Perspectives Psychol Sci* 2013;8:634–50. doi: 10.1177/17456916135041 26173228

[R13] Gratton G, Coles MG, Donchin E. A new method for off-line removal of ocular artifact. *Electroencephalogr Clin Neurophysiol* 1983;55:468–84. doi: 10.1016/0013-4694(83)90135-9 6187540

[R14] Gu Y, Mai X, Luo YJ. Do bodily expressions compete with facial expressions? Time course of integration of emotional signals from the face and the body. *PLoS One* 2013;8:e66762. doi: 10.1371/journal.pone.0066762 PMC3720771 23935825

[R15] Hietanen JK, Astikainen P. N170 response to facial expressions is modulated by the affective congruency between the emotional expression and preceding affective picture. *Biol Psychol* 2013;92:114–24. doi: 10.1016/j.biopsycho.2012.10.005 23131616

[R16] Hietanen JK, Kirjavainen I, Nummenmaa L. Additive effects of affective arousal and top-down attention on the event-related brain responses to human bodies. *Biol Psychol* 2014;103:167–75. doi: 10.1016/j.biopsycho.2014.09.003 25224182

[R17] Hilgard J, Weinberg A, Hajcak Proudfit G et al.. The negativity bias in affective picture processing depends on top-down and bottom-up motivational significance. *Emotion* 2014;14:940. doi: 10.1037/a0036791 PMC4172529 24866528

[R18] Hill SE, DelPriore DJ, Vaughan PW. The cognitive consequences of envy: attention, memory, and self-regulatory depletion. *J Personality Soc Psychol* 2011;101:653–66. doi: 10.1037/a0023904 21639650

[R19] Hofmann W, De Houwer J, Perugini M et al.. Evaluative conditioning in humans: a meta-analysis. *Psychol Bull* 2010;136:390–421. doi: 10.1037/a0018916 20438144

[R20] Hütter M . An integrative review of dual-and single-process accounts of evaluative conditioning. *Nat Rev Psychol* 2022;1:640–53. doi: 10.1038/s44159-022-00102-7

[R21] Kato R, Takeda Y. Females are sensitive to unpleasant human emotions regardless of the emotional context of photographs. *Neurosci Lett* 2017;651:177–81. doi: 10.1016/j.neulet.2017.05.013 28495274

[R22] Koster EHW, Crombez G, Van Damme S et al.. Does imminent threat capture and hold attention?. *Emotion* 2004;4:312–17. doi: 10.1037/1528-3542.4.3.312 15456400

[R23] Krasowski C, Schindler S, Bruchmann M et al.. Electrophysiological responses to negative evaluative person-knowledge: effects of individual differences. *Cognit Affect Behav Neurosci* 2021;21:822–36. doi: 10.3758/s13415-021-00894-w 33846952 PMC8354867

[R24] Leite J, Carvalho S, Galdo-Alvarez S et al.. Affective picture modulation: valence, arousal, attention allocation and motivational significance. *Int J Psychophysiol* 2012;83:375–81. doi: 10.1016/j.ijpsycho.2011.12.005 22226675

[R25] Liang J, Lin H, Jin H. Emotional cues reduce the effects of anticipation violation on ERP responses to facial expressions. *Adv Cognit Psychol* 2024;20:155–66. doi: 10.5709/acp-0426-6

[R26] Lin H, Bruchmann M, Schindler S et al.. Acquisition and generalization of emotional and neural responses to faces associated with negative and positive feedback behaviours. *Front Neurosci* 2024;18, 1399948. doi: 10.3389/fnins.2024.1399948 PMC11334220 39165343

[R27] Lin H, Bruchmann M, Straube T. Altered putamen activation for social comparison-related feedback in social anxiety disorder: a pilot study. *Neuropsychobiology* 2023;82:359–72. doi: 10.1159/000531762 37717563

[R28] Lin H, Liang J. Contextual effects of angry vocal expressions on the encoding and recognition of emotional faces: an event-related potential (ERP) study. *Neuropsychologia* 2019;132:107147. doi: 10.1016/j.neuropsychologia.2019.107147 31325481

[R29] Lin H, Liang J. ERP effects of malicious envy on schadenfreude in gain and loss frames. *Front Human Neurosci* 2021a;15:663055. doi: 10.3389/fnhum.2021.663055 PMC8397463 34456693

[R30] Lin H, Liang J. The effect of malicious envy on schadenfreude when schadenfreude is elicited through social comparisons. *Frontiers in Psychology* 2021b;12:769826. doi: 10.3389/fpsyg.2021.769826 PMC8711567 34966330

[R31] Lin H, Liang J. Working memory load reduces the processing of outcome evaluation involving others but not oneself: event‐related potential evidence. *Psychophysiology* 2021c;58:e13938. doi: 10.1111/psyp.13938 34482549

[R32] Lin H, Liang J. The priming effects of emotional vocal expressions on face encoding and recognition: an ERP study. *Int J Psychophysiol* 2023;183:32–40. doi: 10.1016/j.ijpsycho.2022.11.006 36375630

[R33] Lin H, Liang J. Comparison with others influences encoding and recognition of their faces: behavioural and ERP evidence. *NeuroImage* 2024a;288:120538. doi: 10.1016/j.neuroimage.2024.120538 38342189

[R34] Lin H, Liang J. Competition influences outcome processing involving social comparison: an ERP study. *Psychophysiology* 2024b;61:e14477. doi: 10.1111/psyp.14477 37888488

[R35] Lin H, Schulz C, Straube T. Cognitive tasks during expectation affect the congruency ERP effects to facial expressions. *Front Human Neurosci* 2015a;9:596. doi: 10.3389/fnhum.2015.00596 PMC4623202 26578938

[R36] Lin H, Schulz C, Straube T. Fearful contextual expression impairs the encoding and recognition of target faces: an ERP study. *Front Behav Neurosc* 2015b;9:237. doi: 10.3389/fnbeh.2015.00237 PMC4557081 26388751

[R37] Lin H, Schulz C, Straube T. Effects of expectation congruency on event-related potentials (ERPs) to facial expressions depend on cognitive load during the expectation phase. *Biol Psychol* 2016;120:126–36. doi: 10.1016/j.biopsycho.2016.09.006 27666368

[R38] Lin H, Schulz C, Straube T. Contextual effects of surprised expressions on the encoding and recognition of emotional target faces: an event-related potential (ERP) study. *Biol Psychol* 2017;129:273–81. doi: 10.1016/j.biopsycho.2017.09.011 28939385

[R39] Luck SJ, Hillyard SA. Electrophysiological correlates of feature analysis during visual search. *Psychophysiology* 1994;31:291–308. doi: 10.1111/j.1469-8986.1994.tb02218.x 8008793

[R40] Matyjek M, Kroczek B, Senderecka M. Socially induced negative affective knowledge modulates early face perception but not gaze cueing of attention. *Psychophysiology* 2021;58:e13876. doi: 10.1111/psyp.13876 PMC8459251 34110019

[R41] Mendes WB, Blascovich J, Major B et al.. Challenge and threat responses during downward and upward social comparisons. *Eur J Social Psychol* 2001;31:477–97. doi: 10.1002/ejsp.80

[R42] Moran T, Nudler Y, Bar-Anan Y. Evaluative conditioning: past, present, and future. *Annu Rev Psychol* 2023;74:245–69. doi: 10.1146/annurev-psych-032420-031815 36130066

[R43] Mussweiler T, Rüter K, Epstude K. The man who wasn’t there: subliminal social comparison standards influence self-evaluation. *J Exp Soc Psychol* 2004;40:689–96. doi: 10.1016/j.jesp.2004.01.004

[R44] Righart R, De Gelder B. Context influences early perceptual analysis of faces—an electrophysiological study. *Cereb Cortex* 2006;16:1249–57. doi: 10.1093/cercor/bhj066 16306325

[R45] Righart R, De Gelder B. Rapid influence of emotional scenes on encoding of facial expressions: an ERP study. *Soc Cognit Affect Neurosci* 2008;3:270–78. doi: 10.1093/scan/nsn021 19015119 PMC2566764

[R46] Schindler S, Bruchmann M, Krasowski C et al.. Charged with a crime: the neuronal signature of processing negatively evaluated faces under different attentional conditions. *Psychol Sci* 2021;32:1311–24. doi: 10.1177/0956797621996667 34296955

[R47] Schmader T, Lickel B. The approach and avoidance function of guilt and shame emotions: comparing reactions to self-caused and other-caused wrongdoing. *Motivation Emotion* 2006;30:42–55. doi: 10.1007/s11031-006-9006-0

[R48] Schupp HT, Cuthbert BN, Bradley MM et al.. Affective picture processing: the late positive potential is modulated by motivational relevance. *Psychophysiology* 2000;37:257–61. doi: 10.1017/S0048577200001530 10731776

[R49] Smith RH . Assimilative and contrastive emotional reactions to upward and downward social comparisons. In: Suls J, Wheeler L (eds.), *Handbook of Social Comparison: Theory and Research* Boston, MA, USA: Springer, 2000, 173–200.

[R50] Sugimoto H, Dolcos F, Tsukiura T. Memory of my victory and your defeat: contributions of reward-and memory-related regions to the encoding of winning events in competitions with others. *Neuropsychologia* 2021;152:107733. doi: 10.1016/j.neuropsychologia.2020.107733 33347912

[R51] Suls J, Martin R, Wheeler L. Social comparison: why, with whom, and with what effect?. *Curr Directions Psychol Sci* 2002;11:159–63. doi: 10.1111/1467-8721.00191

[R52] Tor A, Garcia SM. The neuroscience of social comparison and competition. *Cognit Affect Behav Neurosci* 2023;23:920–43. doi: 10.3758/s13415-023-01107-2 37286762

[R53] Valt C, Sprengeler MK, Stürmer B. Feedback processing in the context of social comparison. *Psychophysiology* 2020;57:e13489. doi: 10.1111/psyp.13489 31578749

[R54] van de Ven N, and Zeelenberg M. Envy and social comparison. In: Suls J, Collins RL, Wheeler L (eds.), *Social Comparison, Judgment, and Behavior*. Oxford, England: Oxford University Press, 2020, 226–50.

[R55] Wheeler L, Martin R, Suls J. The proxy model of social comparison for self-assessment of ability. *Personality Soc Psychol Rev* 1997;1:54–61. doi: 10.1207/s15327957pspr0101_4 15647128

[R56] Xu Q, Yang Y, Tan Q et al.. Facial expressions in context: electrophysiological correlates of the emotional congruency of facial expressions and background scenes. *Front Psychol* 2017;8:308212. doi: 10.3389/fpsyg.2017.02175 PMC5733078 29312049

[R57] Zell E, Strickhouser JE, Sedikides C et al.. The better-than-average effect in comparative self-evaluation: a comprehensive review and meta-analysis. *Psychol Bull* 2020;146:118–49. doi: 10.1037/bul0000218 31789535

[R58] Zhang M, Fu Q, Chen YH et al.. Emotional context modulates micro‐expression processing as reflected in event‐related potentials. *PsyCh J* 2018;7:13–24. doi: 10.1002/pchj.196 29297992

